# Mechanisms of Insulin Signaling as a Potential Therapeutic Method in Intestinal Diseases

**DOI:** 10.3390/cells13221879

**Published:** 2024-11-14

**Authors:** Sara Jarmakiewicz-Czaja, Aneta Sokal-Dembowska, Katarzyna Ferenc, Rafał Filip

**Affiliations:** 1 Institute of Health Sciences, Medical College of Rzeszow University, 35-959 Rzeszow, Poland; sjczaja@ur.edu.pl (S.J.-C.); asokal@ur.edu.pl (A.S.-D.); 2 Institute of Medicine, Medical College of Rzeszow University, 35-959 Rzeszow, Poland; kferenc@ur.edu.pl; 3 Department of Gastroenterology with IBD Unit, Clinical Hospital No. 2, 35-301 Rzeszow, Poland

**Keywords:** GLP-1, IBD, insulin, IGF-1, inflammation, TL1A

## Abstract

Gastrointestinal diseases are becoming a growing public health problem. One of them is inflammatory bowel disease (IBD), which includes ulcerative colitis (UC) and Crohn’s disease (CD). The incidence of IBD is increasing in developing countries and declining in developed countries, affecting people of all ages. Researchers have been exploring new treatment options including insulin signaling pathways in the inflammation of the gastrointestinal tract. It seems that a better understanding of the mechanism of IGF-1, GLP-1 and TL1A on the gut microbiota and inflammation may provide new advances in future therapeutic strategies for patients with IBD, but also other intestinal diseases. This review aims to synthesize insights into the effects of GLP, IGF and anti-TL1A on inflammation and the gut microbiota, which may enable their future use in therapy for people with intestinal diseases.

## 1. Introduction

Inflammatory bowel diseases (IBDs), including ulcerative colitis (UC) and Crohn’s disease (CD), are chronic diseases with periods of exacerbation and remission. In UC, inflammation is localized to the rectum and/or colon, involving the mucosa and submucosa [1]. In CD, inflammation can be located along the entire length of the gastrointestinal tract, from the mouth, through the esophagus, stomach, small intestine, large intestine, and into the rectum. However, most often it is localized in the terminal segment of the small intestine and involves the entire thickness of the gastrointestinal wall [2]. In developing countries, there is an increase in the incidence of IBD, whereas in developed countries, there is a stabilization of the cases. Diseases can occur at any age, but the peak incidence occurs between the second and fourth decades of life. The incidence occurs at similar levels in both men and women [3,4].

Despite the many therapeutic agents available, which include glucocorticosteroids, immunosuppressants and biologics, there are patients who do not achieve remission of the disease. Therefore, recently, researchers have been focusing on new pharmaceutical options, which include a mechanism for rectal insulin signaling [5]. The purpose of our review is to summarize the mechanisms of the effects of insulin, glucagon-like peptide (GLP) and insulin-like growth factor (IGF) on inflammation and the gut microbiota to date. As a consequence, it will be possible to discover a new therapeutic option not only for patients with IBD, but also for other intestinal diseases.

## 2. Mechanisms of Intestinal Inflammation

Mechanisms of intestinal inflammation affect each other and are interrelated, including interactions between immune cells, the intestinal microbiota, and the intestinal epithelium [6]. Several mechanisms can be distinguished that predispose to inflammation in the intestines. One of them is an inadequate response of the immune system. One component is immune system cells, such as macrophages, which help the body maintain tissue homeostasis, which is why a link has been observed between abnormal changes in monocytes to macrophages and the occurrence of IBD [7]. Furthermore, macrophages with the M1 phenotype may predispose to IBD, while appropriate therapy that promotes the polarization of M2 macrophages may reduce disease activity [8]. Another type of cell is dendritic cells (DCs), which are found in the intestinal lamina propria that contribute to maintaining intestinal homeostasis [9]. In IBD, colonic DCs show an abnormal immature phenotype; in addition, intestinal mucosa DCs in CD secrete greater amounts of interleukin (IL)-12 and IL-6, and show an increased expression of toll-like receptors (TLR) 4 and TLR2 [10]. Lymphocytes also play an important role in the mechanism of intestinal inflammation. Pro-inflammatory T cells that enter the intestinal tract in IBD, for example, are T helper (Th) 1, which secrete interferon gamma (IFN-γ), which in turn shows the ability to stimulate macrophages to produce and secrete pro-inflammatory cytokines. Another example is Th17, which secretes cytokines and also shows an indirect effect by improving neutrophil recruitment [11,12,13]. In addition, transcription factors and signaling pathways also play an important role in the mechanisms of the inflammatory process, e.g., nuclear factor-κB (NF-κB), signal transducer and activator of transcription 3 (STAT3) and mitogen-activated protein kinase (MAPK) [14,15,16].

### 2.1. Factors Related to the Intestinal Microbiota That Affect the Maintenance of Homeostasis in the Intestinal Tract

One of the factors predisposing to IBD is intestinal dysbiosis. A decrease in microbial diversity and a change in quality, an increase in the number of pathogenic microorganisms and a decrease in the commensal microbiota affect the appearance of a number of changes in the normal functioning of the intestinal barrier and the maintenance of intestinal homeostasis [17]. There are several factors that regulate the inflammatory process that occurs in the intestines. One of them is the regulation of the immune system. Certain bacterial species have been linked to Th cell development and Treg cell induction [18]. Lactobacillus rhamnosus GG (LGG), on the other hand, can stimulate Th17/Treg balance in the gut [19]. Macrophages also respond to stimuli from the gut microbiota. PGE2 + macrophages are crypt niche cells that increase Wnt/β-catenin signaling in intestinal stem cells via prostaglandin E2 (PGE 2) receptors [20]. Another factor against the appearance of inflammation in the intestine is the proper functioning of the intestinal barrier. It is crucial to preventing the passage of pathogenic microorganisms into the body. Factors that determine the proper function of the intestinal barrier include tight junctions. These are transmembrane proteins that connect neighboring intestinal cells; they include, for example, claudins and zonula occludens [21]. In addition, the protective barrier is formed by a layer of mucus, which consists mainly of water, lipids, electrolytes, and proteins. Some intestinal microorganisms can affect the regulation of the mucus barrier (*A. muciniphila*). They can increase mucus secretion and the thickness of the mucus layer [22]. Another factor is pattern recognition receptors (PRRs), which recognize structures in pathogens called pathogen-associated molecular patterns (PAMPs) [23]. Examples include TLR4, which recognizes lipopolysaccharides (LPS), components of bacterial cell membranes, and TLR5, which recognizes flagellin, a protein that builds bacterial threads [24,25]. Furthermore, a receptor belonging to the NOD-like receptor (NLR) family, i.e., nucleotide-binding oligomerization domain-containing protein 2 (NOD2), participates in maintaining normal intestinal homeostasis through the regulation of the immune system. NOD2 deficiency is associated with reduced antimicrobial activity by Paneth cells, and an imbalance of the intestinal microbiota can occur [26].

### 2.2. Receptor-Mediated Insulin Signaling

More recently, it has been shown that metabolism is involved in the regulation of immunity [27]. Insulin is a key hormone that is involved in the regulation of glucose, but also fat and protein metabolism. It acts through the insulin receptor (IR) to influence the regulation of nutrient uptake and storage in liver tissues, adipose tissue and muscle. IR has been shown to exist as two isoforms: IR-A and IR-B. The difference between these isoforms is the exclusion (IR-A) or inclusion (IR-B) of exon 11. This exon encodes a 12-amino acid region that is located at the C-terminus of the subunit that binds the α IR ligand [28]. IR-A has a high affinity for insulin and IGF-2, and about a ten-times-lower affinity for GF-1. In contrast, IR-B binds insulin, but has a much lower affinity for insulin-like growth factor (IGF)-1 and IGF-2 [28]. Energy storage by insulin is mediated by the phosphotidyl-inositol-3 kinase (PI3K) and MAPK pathways. This results in the activation and phosphorylation of insulin receptor substrates (IRSs) [29]. Insulin receptor signaling enhances nutrient uptake through T cells [30].

IR appears to be expressed in the intestinal epithelium, although its role in growth or function is not clearly defined [28]. In addition, the insulin pathway exhibits regulatory effects in mucosal immunology [31]. Zhang et al. showed that high glucose intake promotes Th17 lymphocyte differentiation. This occurs through a mechanism of transforming growth factor-beta (TGF-β) activation via an increase in reactive oxygen species (ROS) in T lymphocytes. In addition, high amounts of glucose promoted the exacerbation of autoimmunity in mice with artificially induced colitis [32]. Yassin et al. in their study presented INSR in the intestinal epithelium as a possible therapeutic target for IBD. The researchers suggest that INSR may accelerate tissue regeneration and increase the resistance of the EIC to damage from inflammation. These actions appear to be facilitated by increased expression of cytoprotective proteins such as Car3 [33]. In contrast, in a study on mice with dextran sulfate sodium (DSS)-induced intestinal inflammation, Li et al. showed that the activation of the insulin pathway increased the expression of enhancer of zeste homolog 2 (EZH2) in mucosal T cells during inflammation. This promoted the differentiation of tissue-resident memory (TRM) T cells, but also upregulated intestinal intraepithelial lymphocytes (IELs) and inflammatory cytokines. Consequently, it promoted the exacerbation of colitis during the inflammatory phase [5]. Due to the discrepancy in some of the findings, further research is needed to clarify and define the role of IR in intestinal diseases

## 3. Regulation of Inflammation in the Intestines

### 3.1. The Role of Insulin in Regulating Inflammation

According to Makhijani et al., disturbed insulin-dependent metabolic homeostasis leads to immune dysfunction, and sensitization to the action of this hormone may bring benefits in reducing inflammation [34]. Systemic inflammation can lead to the development of many diseases, including cardiovascular diseases, cancer, non-alcoholic liver disease and diabetes. Due to the common nature of the problem, inflammatory diseases have been considered a more important cause of death worldwide [35]. Hyperglycemia, an early complication of diabetes, can also indue inflammatory programming of macrophages [36,37]. Hyperglycemia in type 2 diabetes (T2DM) may occur as a result of insulin resistance and inappropriate insulin secretion, and glycemic control can be achieved by administering insulin in an appropriate dose [37]. It is believed that insulin may play an important immunomodulatory and anti-inflammatory role in the body [38].

As available data indicate, insulin can exert homeostatic and anti-inflammatory effects in the human body. However, the molecular mechanism by which insulin can reduce inflammation in the body is still not fully understood. There are likely several potential mechanisms by which insulin may reduce inflammation in the body. One of them is its ability to lower blood glucose levels [39]. Hyperglycemia in diabetes may induce toxicity due to the greater flow of glucose through the glycolytic pathway [40]. Additionally, hyperglycemia caused by oxidative stress and cytokine release leads to the expression of cell adhesion molecules on activated endothelial cells in diabetic patients [41]. As a result of oxidative stress, mitochondria become damaged and a number of factors are activated, including the activation of TLRs, which may lead to the dysfunction of pancreatic β-cells [42]. Previous findings have already shown that insulin can inhibit the expression of TLRs at the transcriptional level, probably through its inhibitory effect on the PU.1 transcription factor [43].

It has also been shown that the concentrations of cell adhesion molecules (intercellular adhesion molecule 1—ICAM; vascular cell adhesion molecule 1—VCAM; E-selectin) positively correlate with an increase in the level of glycated hemoglobin (HbA1c) in young people with type 2 diabetes and are associated with blood pressure and microalbuminuria, which are markers of vascular damage [44]. In patients with type 2 diabetes who were introduced to intensive insulin therapy using continuous subcutaneous infusion, a reduction in pro-inflammatory cytokines was observed. Regardless of the initial HbA1c level, a reduction in interleukin-6 receptor (IL-6R), regulated upon activation normal T-cell expressed and secreted (RANTES) and ICAM-1 levels was observed in all groups. These cytokines were significantly involved in the tumor necrosis factor (TNF) signaling pathway [45]. ICAM-1 is a key molecule in the progression of inflammation and leads to endothelial cell damage [46]; therefore, inhibiting ICAM-1 release through insulin supply may likely prevent the development of complications.

It is worth noting that lowering glucose levels is associated with a lower number of advanced glycation end products (AGEs) and a reduction in the activity of nuclear factor kappa-light-chain-enhancer of activated B cells (NF-κB) via RAGE (receptor for AGEs), and thus reducing inflammation [47]. Insulin can suppress LPS/TLR 4signals in leukocytes via the mTORC2-Akt-FoxO signaling axis [48]. It is likely that the reduction in FOXO1 and TLR transcription may serve to suppress insulin-mediated inflammation [34].

Another interesting effect is the effect of insulin on the production of nitric oxide (NO) from the vascular endothelium, which, through increased blood flow, increases glucose uptake in skeletal muscles [49]. In diabetic patients, vascular resistance to NO develops, and insulin supply may reduce this resistance by reducing oxidative stress and peroxygen production [50]. In patients with IBD, the induction of endogenous NO production by enterocytes with supplements may result in improved epithelial integrity and the amelioration of colitis [51].

A study by Huang et al. conducted in an animal model showed that insulin can alleviate encephalopathies in sepsis by inhibiting astrocyte NFκB and microglial MAPK [52]. A study by Chang et al. showed that insulin is a key regulator of NLPRP3 inflammasome activation. It is likely that insulin exerts its anti-inflammatory effects by attenuating ASC speckle formation to reduce inflammasome activation and reduce pro-inflammatory cytokine secretion in an INSR- and IGF1R-dependent manner. Moreover, insulin administration reduced the production of IL-1β by THP-1 cells [38].

### 3.2. Reducing Inflammation in the Gut by Influencing the Gut Microbiota

Different glycemic states can alter intestinal physiology [53]. A state of elevated blood glucose may cause increased intestinal permeability through glucose transporter 2 (GLUT2)-dependent transcriptional reprogramming of the intestinal epithelium and alteration of tight junction integrity and adhesion [54]. In an in vitro study, Dubois et al. showed that high glucose levels induce morphological and functional changes such as increased permeability, reduced mucus secretion and increased alkaline phosphatase activity. The authors of the study emphasized that the main therapeutic goal in maintaining intestinal health is adequate glycemic control [55].

There are many data indicating the relationship between impaired glycemia and intestinal dysbiosis [56,57,58,59,60]. In a study on an animal model conducted by Wang et al., a positive effect of intensive insulin therapy on the improvement of intestinal morphological parameters was noted, including its length, villus height, microvilli length and crypt depth. Moreover, a beneficial effect in modulating the intestinal microbiota at various taxonomic levels has been demonstrated. *Bacterioidetes* decreased, while *Firmicutes actinobacteria* and *Deferribacteres* increased significantly [61].

An analysis of the fecal microbiota of pregnant women suffering from gestational diabetes (GDM) showed a higher content of Clostridiales, Lactobacillus and Bacteroidetes. Then, after insulin treatment, a higher Firmicutes/Bacteroidetes ratio was found compared to women who controlled GDM with diet, which was also observed in meconium and the first feces of newborns [62].

**Figure 1:**
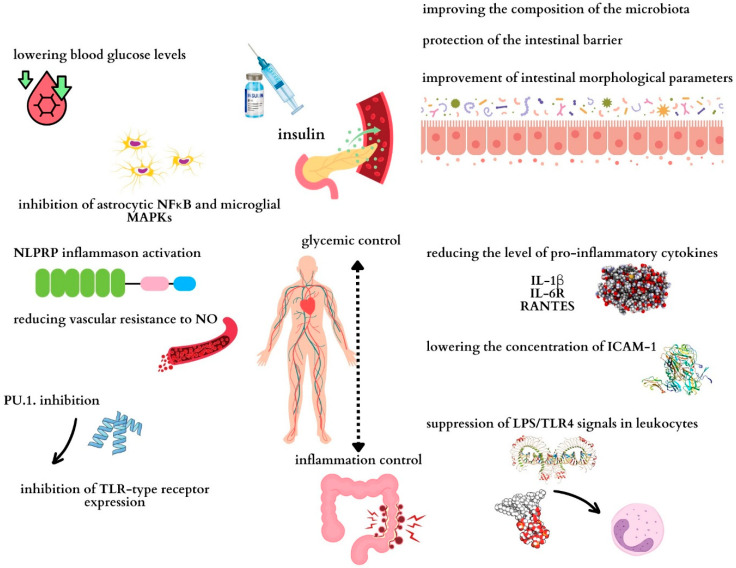
Potential mechanisms through which insulin may exert anti-inflammatory effects on the intestine. Maintaining proper glycemia determines the control of inflammation in the body and therefore in the intestines. NFκB: nuclear factor kappa-light-chain-enhancer of activated B cells; ICAM-1: intercellular adhesion molecule 1; NO: nitric oxide; TLR4: Toll-like receptor 4; LPS: lipopolysaccharide; IL-1β: interleukin 1 beta; IL-6R: interleukin-6 receptor; RANTES: regulated on activation, normal T-cell expressed and secreted.

A summary of the anti-inflammatory effects of insulin is presented in Figure 1.

### 3.3. Other Factors That Reduce Inflammation in the Gut

#### 3.3.1. A High-Fiber Diet

A diet rich in dietary fiber has a positive effect on the composition of the intestinal microbiota and may reduce inflammation in the intestines. Fiber metabolism also leads to the release of feluric acid, which has antioxidant and anti-inflammatory properties [63].

The fermentation of whey protein with a glycolic acid and pentozophosphorus acid results in microbiological fermentation allowing the extraction of sugar cane sugar (SCFA). Bacteria involved in the development of SCA include *Bacteroides* spp. *Prevotella* spp., and *Akkermansia muciniphila* [64,65]. Among the SCAs, acetic acid, propionic acid and butyric acid stand out, but butyric acid is considered to have the greatest anti-inflammatory role in the body. The bacteria are from the *Firmicutes* type, including *Roseburia*, *Eubacterium rectale* and *Faecalibacterium prausnitzii*. Butyrate inhibits Th17 cell differentiation and promotes Treg differentiation and secretion of IL-10 and the secretory IgA [65]. It is probably the case that butyrate can improve insulin sensitivity and regulate glucose metabolism. Butyrate’s effect on insulin signaling is related to its metabolism. In a study by Rios-Morales et al., butyrate increased the levels of proteins and metabolites involved in butyrate oxidation itself. The inhibition of butyrate oxidation increased its availability in the body, and its higher concentration led to histone deacetylase inhibition leading to improved insulin sensitivity [66]. HDAC can regulate glucose metabolism, insulin release expression and also insulin signaling [67]. Additionally, SCFAs can affect insulin signaling by affecting β-cells and intestinal cells thereby altering the function of certain proteins [68].

Studies indicate that SCFAs act on G protein-coupled receptors (GPCRs): GPR43/FFAR2 and GPR41/FFAR3. These receptors are found in many tissues in the body, including pancreatic β-cells [69].

FFAAR2 is present in pancreatic islet α and β cells as well as intestinal enteroendocrine cells, and FFAR3 expression additionally occurs on immune cells, intestinal neurons and sympathetic ganglia, among others [66].

The role of SCFAs in reducing inflammation, improving insulin signaling and glucose metabolism still needs to be thoroughly investigated.

#### 3.3.2. Dietary Supplements

Supplements used to reduce inflammation in the intestine include probiotics, butyrate, lactoferrin, palmitoylethanolamide, phosphatidocholine, silymarin and omega 3 acids. It is believed that these substances, in addition to their anti-inflammatory effect, can support maintaining the microbial balance in the intestines and strengthen the barrier function of the mucosa (Table 1) [70].

#### 3.3.3. Physical Activity

A recent meta-analysis of 10 observational studies found that physical activity is inversely associated with the risk of developing IBD [77]. A sedentary lifestyle may cause a Th1/Th2 imbalance, which directly affects the secretion of pro-inflammatory and anti-inflammatory cytokines [78]. Moderate exercise has been associated with a reduction in the levels of cytokines such as IL-1 and TNF-α and an increase in anti-inflammatory factors IL-6, IL-10 and IL-1ra (interleukin-1 receptor antagonist) released by myokines [79].

#### 3.3.4. Adequate Amount of Sleep

Disturbances in circadian rhythms have been associated with worsening of the severity of colitis. It is likely that improving the quality and treatment of sleep disorders may help to alleviate inflammation in the gut [80]. Sleep deprivation may lead to cognitive impairment and activation of the TLR4/NF-κB signaling pathway and deterioration of the intestinal microbiota, thus leading to inflammation [81]. Bermingham et al. showed that social jet lag caused by, among others, shift work and the lack of a fixed sleep rhythm can lead to intestinal dysbiosis and inflammation [82]. Ensuring good quality and duration of sleep seems to be important to reduce intestinal inflammation.

## 4. Effects of Insulin-like Growth Factor 1 (IGF-1) on Intestinal Inflammation and Gut Microbiota

IGF is a large family with three ligands (IGF-1, IGF-2, insulin) and two receptors (IGF-1 (IGF-IR) and IGF-2 (IGF-IIR)). In the gastrointestinal tract, the IGF system exists as a paracrine, endocrine and autocrine regulator for cell proliferation, survival and apoptosis. In addition, IGF is important in carbohydrate and protein metabolism, but also affects energy balance. Most of the circulating IGF-1 is synthesized in the liver through the regulation of growth hormone (GH) [83]. So far, it has been shown that the most common metabolites of the gut microbiota are short-chain fatty acids, serotonin, polyamines and ATP. However, recent studies have documented that the microbiota also affects insulin-IGF-1 levels [84,85,86]. Low IGF-1 levels appear to be associated with the induction of bacterial translocation growth [87]. In addition, it has been observed that the use of antibiotic therapy in mice reduces serum IGF-1. As a consequence, there is a decrease in bone mass. In contrast, after treatment with SCFAs, the amount of IGF-1 returns to normal, but also bone mass returns to levels like those in mice not treated with antibiotics. The study authors suggest that it is SCFAs that may be involved in the induction of IGF-1 by the gut microbiota [88].

Chena et al. showed that IGF-1 contributes to mucosal regeneration through the β-arrestin2-mediated extracellular signal-associated kinase signaling pathway [89]. Xu et al. showed that the intravenous injection of human tonsil-derived MSCs (T-MSCs) had an effect on alleviating colitis. Moreover, the analysis showed that administration of T-MSCs increased endogenous IGF-1 in treated mice. In contrast, the administration of IGF-1 receptor inhibitors reduced the therapeutic functions of T-MSCs. Additional stimulation of IGF-1 had the effect of reducing apoptosis and promoting cell proliferation. The authors suggest that acting on endogenous IGF-1 secretion can be used as a therapeutic strategy by maintaining the integrity and promoting the regeneration of the colonic epithelium [90]. In addition, another study showed that MSCs supplemented with IGF-1, among others, induced cell cycle regulation of intestinal stem cells and induced a balance of anti- and pro-inflammatory cytokines. The result was a mitigation of intestinal damage [91]. Similarly, another study using MSCs and IGF-1 observed that IGF-1 maximized the therapeutic effect of MSCs by attenuating inflammation, but also promoting colon regeneration [92]. An analysis of parenteral nutrition in rats showed that both GH and IGF-1 administration reduced total body weight loss and nitrogen excretion. In addition, they induced an increase in protein synthesis. In addition, the results showed that GH action increased IGF-1-mRNA levels in the liver and IGF-1 levels in plasma. In contrast, IGF-1 action increased IGF-1 in plasma, but did not change IGF-1-mRNA levels in the liver [93]. In another study, an attempt was made to treat experimental colitis through the use of GH. The process was found to increase IGF-1 levels and body weight in mice, but without affecting colitis [94]. Bohin et al. in a study in mice, showed that IGF-1 signaling through mTORC1 activation potentially induced regeneration of intestinal crypts [95]. Another study showed that the action of IGF-1 combined with fibroblast growth factor 2 (FGF-2) can increase cell diversity in small intestinal organoids in mice [96]. One study confirmed that IGF-1 levels were reduced in patients. In contrast, prednisolone treatment affected its increase [97]. Sipos et al. showed that CD patients exhibit increased expression of the insulin-like growth factor 1 receptor (IGF1R) signaling pathway in submucosal fibroblast cells and subserosal adipocytes [98]. It appears that IGF-1 release may be the main therapeutic mechanism in the treatment of experimental colitis with ghrelin [99]. However, studies on the mechanisms of IGF-1 in IBD patients are still lacking.

## 5. Effects of Glucagon-like Peptide-1 (GLP-1) on Intestinal Inflammation and Gut Microbiota

Glucagon-like peptide-1 (GLP1) is an incretin hormone that controls glycemia, slows intestinal motility, and shows receptor expression in various types of mucosal cells. It is produced in the L cells of the small intestine and large intestine, while it is released after nutrients are delivered to the intestinal lumen [100].

The effects of GLP1 on the gut are multidirectional and can affect, among other things, the integrity of the intestinal barrier. In their study, Anbazhagan et al. showed that GLP-1-SSM administered as an injection can reduce intestinal mucosal inflammation and the incidence of diarrhea in patients with IBD [101]. It can also reduce intestinal permeability and intensify the mucosal healing process [102]. In another study, Funayama et al. observed that a centrally administered GLP-1 analog showed the ability to improve colon permeability [103]. GLP1 can also improve intestinal barrier function by modulating the intestinal microbiota and stimulating crypt cell division [104]. GLP-1 receptor agonists up-regulate chemokine ligand 20 (CCL20), mucin 5b (MUC5) and IL-33, with which they show the ability to influence the maintenance of homeostasis in the gut [105]. Ebbesen et al. also indicate that GLP1 may exhibit regenerative capacity of damaged epithelial barriers [106]. The GLP-1R agonist (liraglutide) had a beneficial effect on TJ proteins (zonula occludens-1 (ZO-1), occludins) [107]. Arvanitakis et al. came to a similar conclusion in their review that by administering glucagon-like peptide 1 receptor agonists (GLP-1 RAs) to patients with IBD, the benefits of therapy can be maximized, among other things, by maintaining and adequately functioning TJ [108]. In another study, similarly, liraglutide showed the ability to reduce endothelial nitric oxide synthase (eNOS) expression and to recover normal TJ protein function [109]. The protection of the intestinal mucosa in the context of IBD is very important, as patients experience damage to the intestinal barrier, which predisposes pathogenic microorganisms to enter the body, thus causing inflammation [6]. Furthermore, GLP1 and its agonists can reduce anti-inflammatory cytokine production by, among other things, reducing NF-κB phosphorylation [110]. Amato et al. also showed that the expression of IL-6, IL-8, IL-1β and TNF-α decreased after teduglutide treatment [111]. In addition to reducing pro-inflammatory cytokine secretion, liraglutide therapy also attenuated neutrophil infiltration into the intestine [112]. GLP-1 also shows the ability to reduce the expression of CD69 and CD11b and the production of IL-4 and IL-8 by LPS-induced eosinophils [113]. GLP 1 also affects changes in the gut microbiota. In their paper, Everard et al. present a link between bacterial metabolites and the triggering of GLP1 secretion. However, they emphasize that certain bacteria, e.g., Bifidobacterium spp, can modulate this relationship both indirectly and directly [114]. In Angelini et al., similar conclusions were reached by the gut microbiota (SCFAs) modulating the activity of enteroendocrine cells, increasing, for example, GLP1 secretion [115]. On the other hand, SCFAs, such as butyric acid, show anti-inflammatory properties [116].

## 6. Tumor Necrosis Factor-like Ligand 1A (TL1A)

Tumor necrosis factor (TNF)-like cytokine 1A (TL1A) belongs to the TNF family. It is a type 2 transmembrane protein first described in 2002. TL1A expression is increased in macrophages and dendritic cells [117]. It is secreted by cells that present the antigen after stimulation [118]. It can regulate inflammation and intestinal homeostasis through the TL1A/DR3/DcR3 pathway [119]. Anti-TL1A treatment targets TL1A (Tumor Necrosis Factor-like ligand 1A), which regulates mucosal defense in the intestines, and therefore inflammation, and is involved in the pathogenesis of IBD [120]. Therefore, the human immunoglobulin G1 monoclonal antibody (anti-TL1A antibody) is a potential therapeutic agent for moderate to severe UC [121]. The use of such therapies has been shown to potentially inhibit endogenous TL1A activity [122]. Furthermore, TL1A and its functional death receptor 3 (DR3) may play a role in intestinal fibrosis during ongoing inflammation [123]. DR3 signaling directly affects fibroblasts, influencing activation and migration, and ultimately intestinal fibrosis [124]. Therefore, the modified stable ligand TL1A shows the ability to stimulate T cells by specific binding to DR3 [125]. Jacob et al. also indicate that intestinal fibrosis mediated by TL1A may depend on the intestinal microflora [126]. Bamias’ work shows that TL1A and DR3 enhance Th1, Th2 and Th17 responses [127]. Therefore, the anti-TL1A antibody can alleviate intestinal fibrosis [128]. A study by Yang et al. in animal models showed that TL1A disrupts intestinal epithelial barrier function and affects TJ expression regulation [129]. In another article, the authors found that the activation of DR3 signaling results in loss of ILC3, which is associated with increased inflammation [130]. Anti-TL1A treatment is a promising therapeutic strategy.

## 7. Conclusions

Interactions between immune system cells, intestinal microbiota and the intestinal epithelium are important mechanisms of IBD pathogenesis. Recognition of the complex mechanisms of IBD pathogenesis and potential therapeutic interventions, including the effects of insulin, IGF-1, GLP-1 and TL1A on inflammation and intestinal microbiota, may contribute to the development of more effective therapeutic strategies for patients with inflammatory bowel diseases. There is research suggesting that insulin may exert anti-inflammatory effects by reducing blood glucose levels and regulating signaling pathways related to the immune response. Furthermore, insulin can influence the composition and diversity of microorganisms in the intestines. In turn, the use of GLP-1 receptor agonists may improve the function of the intestinal barrier, and IGF-1 may promote the regeneration of the intestinal epithelium and reduce inflammation. Treatment with anti-TL1A antibodies also seems to be a promising strategy in patients with moderate to severe ulcerative colitis. Further in vitro and clinical studies may be helpful to better understand the role of these factors in maintaining intestinal homeostasis and reducing inflammation in IBD patients.

## Figures and Tables

**Table 1:** The influence of selected dietary supplement on the reduction in inflammation.

Dietary Supplements	Effect on the Intestines
Probiotics	Restoration of epithelial barrier function [70]; Mainly beneficial effects from the use of *Lactobacillus*—protection of the small intestine by increasing microbial diversity, expression of proteins related to homeostasis and strengthening the integrity of the immune system [71]; Maintenance of the integrity of the cellular structure and maintains cell protein stability [71].
Butyrate	Inhibition of interleukin (IL)-12 production by suppressing IL-12p40 and IL-12p35 mRNA accumulation [70]; Increase in the release of IL-10 in human monocytes induced by *S*. *aureus* [70]; Reduction in oxidative stress, modulation of intestinal permeability and protection of the colon barrier [70].
Lactoferrin	Regulation of the immune response of the intestinal mucosa [72]; Restoration of tight junction morphometry and blocking of caspase-3 cleavage [72]; Decreased secretion and expression of TNF-α, IL-6, IL-8, NF-κB genes [72]; Increase in intestinal epithelial cell proliferation, cytokine production and immune cell function [72].
Palmitoylethanolamide	Modulation of intestinal glial cells—counteracting motor dysfunctions and intestinal inflammatory processes [73]; Inhibition of the NLRP3/IL-1β inflammasome pathways [73]; Promotion of macrophage polarization towards an M2-type anti-inflammatory phenotype [73]; Inhibition of the NF-κB p65 pathway and the release of pro-inflammatory cytokines [73].
Phosphatidecholine	Inhibition of pro-inflammatory signaling in phagosome model systems derived from macrophages [70].
Silymarin	Inhibitory effect on TNF-α, interferon, interleukin-2 and iNOS [74]; Inhibition of neutrophil migration towards the site of inflammation [74]; Inhibition of the functions of prostaglandins, leukotrienes, Kupffer cells and NF-κB factor [74].
Omega-3 acid	AA, LA, EPA and DHA acids can lead to the production of substances known as oxylipins [75]; Reduction in the level of CRP and homocysteine [75]; Decreased expression of genes involved in inflammatory cascades, fibrinolysis, leukocyte adhesion and blood coagulation [76].

TNF-α: tumor necrosis factor-α; CRP: C-reactive protein; NF-κB: nuclear factor kappa-light-chain-enhancer of activated B cells; iNOS: nitric oxide synthase; AA: arachidonic acid; LA: linoleic acid; EPA: eicosapentaenoic acid; DHA: docosahexaenoic acid.

## Data Availability

No new data were created or analyzed in this study. Data sharing is not applicable to this article.
